# The Relationship between Peripheral Nerve Conduction Velocity and Ophthalmological Findings in Type 2 Diabetes Patients with Early Diabetic Retinopathy

**DOI:** 10.1155/2018/2439691

**Published:** 2018-02-20

**Authors:** Azusa Ito, Hiroshi Kunikata, Masayuki Yasuda, Shojiro Sawada, Keiichi Kondo, Chihiro Satake, Kazuki Hashimoto, Naoko Aizawa, Hideki Katagiri, Toru Nakazawa

**Affiliations:** ^1^Department of Ophthalmology, Tohoku University Graduate School of Medicine, Sendai, Japan; ^2^Department of Retinal Disease Control, Tohoku University Graduate School of Medicine, Sendai, Japan; ^3^Department of Metabolism and Diabetes, Tohoku University Graduate School of Medicine, Sendai, Japan; ^4^Department of Advanced Ophthalmic Medicine, Tohoku University Graduate School of Medicine, Sendai, Japan; ^5^Department of Ophthalmic Imaging and Information Analytics, Tohoku University Graduate School of Medicine, Sendai, Japan

## Abstract

**Purpose:**

Nerve conduction velocity (NCV) is an indicator of neuronal damage in the distal segment of the peripheral nerves. Here, we determined the association between NCV and other systemic and ocular clinical findings, in type 2 diabetes patients with early diabetic retinopathy (DR).

**Methods:**

This study included 42 eyes of 42 type 2 diabetes patients (median age: 54 years) with no DR or with mild nonproliferative DR. Standard statistical techniques were used to determine associations between clinical findings.

**Results:**

Sural sensory conduction velocity (SCV) and tibial motor conduction velocity (MCV) were significantly lower in mild nonproliferative DR patients than patients with no DR (*P* = 0.008 and *P* = 0.01, resp.). Furthermore, logistic regression analyses revealed that sural SCV and tibial MCV were independent factors contributing to the presence of mild nonproliferative DR (OR 0.83, *P* = 0.012 and OR 0.69 *P* = 0.02, resp.). Tibial MCV was correlated with choroidal thickness (CT) (*P* = 0.01), and a multiple regression analysis revealed that age, tibial MCV, and carotid intima-media thickness were independent associating factors with CT (*P* = 0.035, *P* = 0.015, and *P* = 0.008, resp.).

**Conclusions:**

Our findings suggest that reduced NCV may be closely associated with early DR in type 2 diabetes patients. Thus, reduced nerve conduction is a potential early biomarker of DR.

## 1. Introduction

Diabetes mellitus affects over 350 million people worldwide [1], and approximately one-third of these people are also affected by diabetic retinopathy (DR), or will go on to develop it. Thus, DR has become a leading cause of visual disability among working-aged adults in both developing and developed countries [2]. Diabetes can cause vision-threatening complications, including macular ischemia, diabetic macular edema (DME), and neovascularization, that is, proliferative DR (PDR), even with advances in therapies [3, 4]. Antivascular endothelial growth factors are a common strategy to treat DME, but the disease often recurs, and the clinical success of this treatment is affected by significant numbers of nonresponders [5]. Thus, the best strategy is to prevent the development of PDR and DME in patients with diabetes. Therefore, it is important to understand the pathogenesis of PDR and DME and to find clinically useful new biomarkers of early DR [6, 7].

DR is one of the three major microvascular diabetic complications, together with neuropathy and nephropathy. These complications, all of which are caused by diabetic microvascular disturbances, are generally thought to arise sequentially: neuropathy occurs first, followed by retinopathy, and finally nephropathy. Indeed, diabetic peripheral neuropathy has been observed during the early, impaired glucose tolerance period of diabetes [8, 9]. Moreover, neuronal damage in the distal segment of the peripheral nerves is associated with objective measurements of neuropathy, such as nerve conduction velocity (NCV). In patients with diabetes, retinal neuroglial dysfunction occurs before ophthalmoscopically visible signs of retinopathy (i.e., vascular changes) are seen. This raises the possibility that NCV may be a biomarker of diabetes progression, including ocular complications, in the earliest stages. However, the association between NCV and the severity of retinopathy, as assessed by optical coherence tomography (OCT) findings, has not yet been fully investigated. Furthermore, the relationship between NCV and OCT findings, particularly choroidal thickness (CT), remains unclear [10–14].

The development of OCT has enabled ophthalmologists to quickly, easily, and noninvasively measure CT, as well as central macular thickness (CMT). Recent reports have shown that alterations in CT are associated with the pathogenesis of various ocular diseases, including DR [15–19]. However, the association of CT with NCV is still unclear, particularly in eyes with early DR, that is, those with no DR (NDR) or with mild nonproliferative DR (NPDR) [20]. Thus, in this study, we determined the association between systemic clinical findings, including NCV, and ocular clinical findings, including measurement of retinal structure, in type 2 diabetes patients with early DR, in order to search for new and early biomarkers of DR.

## 2. Materials and Methods

### 2.1. Setting and Design

This was an institutional, cross-sectional, nonrandomized, observational case series.

### 2.2. Patients

All subjects had type 2 diabetes with either NDR or mild NPDR [20] and were observed at the Tohoku University Hospital. The subjects underwent a baseline ophthalmic examination, including measurement of visual acuity and intraocular pressure (IOP), a slit lamp examination, and a fundus examination.

The inclusion criteria were diabetes with hemoglobin A1c (HbA1c) > 6.5% and ongoing pharmacological treatment for diabetes. The exclusion criteria were the presence of pancreatogenic diabetes (type 3c diabetes), hepatic diabetes, gestational diabetes, secondary diabetes from endocrine disease or type 1 diabetes; ongoing hemodialysis; current malignant, inflammatory disease or chronic respiratory disease; age-related macular degeneration; glaucoma; retinal diseases other than DR; and a history of intraocular surgery.

The severity of DR was evaluated in the right eye of each patient by the same experienced ophthalmologist with indirect ophthalmoscopy and slit-lamp biomicroscopy of the posterior segment with a +90 D lens (Volk Optical Inc., Mentor, Ohio, USA) according to the Early Treatment of Diabetic Retinopathy Study (ETDRS) criteria [20, 21].

The institutional review board of the Tohoku University Graduate School of Medicine approved this study. Informed consent was obtained from each patient for his or her participation in the research (University Hospital Medical Information Network; UMIN Study ID number: UMIN000023859), and the research was conducted according to the provisions of the Declaration of Helsinki, 1995 (as revised in Edinburgh, 2000).

### 2.3. Main Outcome Measure

Standard statistical techniques were used to determine associations between clinical findings, including diabetes duration, creatinine, blood levels of HbA1c, CT in OCT scans, intima-media thickness (IMT) in ultrasound B-scans of the right common carotid artery, and NCV.

### 2.4. Measurement of Physical and Ophthalmological Findings

Systolic blood pressure (SBP) was measured after the patients had rested in a sitting position for 10 min. Measurements were made in the left brachial artery at the height of the heart with an automated blood pressure monitor (HEM-759E, Omron Corporation, Kyoto, Japan). The subjects fasted 12 hours before collection of the blood samples. HbA1c, total cholesterol, and creatinine were measured with automated standardized laboratory techniques. IMT was measured in ultrasound B-scans of the common carotid artery, made with the ProSound F75 (Hitachi-Aloka, Tokyo, Japan). Ophthalmological examinations included fundus photography and measurement of visual acuity, IOP, and spherical equivalent (SE). This study used 3D-OCT (Topcon 3D OCT-2000, Topcon, Tokyo, Japan) to measure CMT and enhanced depth imaging (EDI) OCT (OCT Spectralis, Heidelberg Engineering, Germany) to manually measure subfoveal CT. All included OCT data had a minimum image quality > 35 for 3D-OCT and a minimum quality score > 20 for EDI OCT. The eye of each patient with the worse grade of retinopathy was included. If the grade was similar in both eyes, the right eye was selected.

### 2.5. Measurement of Nerve Conduction Velocity

NCV was measured with the MEB-2312 device (Nihon Kohden Corporation, Tokyo, Japan). The types of NCV measured in this study were median motor conduction velocity (MCV), median sensory conduction velocity (SCV), tibial MCV, and sural SCV.

### 2.6. Statistical Analyses

Variables were expressed as the median (interquartile range (IQR)). The Mann-Whitney *U* test and chi-square test were used to evaluate differences in clinical findings in the NDR and mild NPDR groups. Spearman's rank correlation test was used to estimate relationships between variables. Logistic regression analysis was used to determine the significance of the presence of DR in the patients. Separate multiple linear regression analyses were performed to analyze each independent variable potentially affecting CT. All statistical analyses were performed with the R software (version 3.2.0, R core team). Differences were considered significant at *P* < 0.05.

## 3. Results

**Figure 1:**
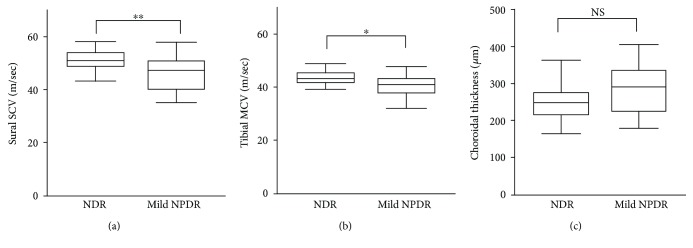
Nerve conduction velocity and choroidal thickness in early diabetic retinopathy. (a) Sural sensory conduction velocity (SCV) was lower in mild nonproliferative diabetic retinopathy (NPDR) patients than in no diabetic retinopathy (NDR) patients (*P* = 0.008). (b) Tibial motor conduction velocity (MCV) was lower in patients with mild NPDR than in patients with NDR (*P* = 0.01). (c) choroidal thickness was higher in patients with mild NPDR than patients with NDR, but with no significant difference (*P* = 0.10). Mann-Whitney *U* test: ^∗^*P* < 0.05 and ^∗∗^*P* < 0.01; NS: not significant.

The clinical characteristics of the diabetes patients are shown in Table 1. This study included 42 patients with type 2 diabetes (26 male, 16 female; median age: 54 years; age range: 27–61). There were no differences in age, sex, SBP, creatinine, total cholesterol, VA, SE, IOP, HbA1c, carotid IMT, or CT in the NDR and mild NPDR groups (25 and 17 patients, resp.) (Table 1 and Figure 1(c)). However, diabetes duration was significantly longer (*P* = 0.04) and CMT was significantly higher (*P* = 0.01) in the mild NPDR group than in the NDR group (Table 1). Furthermore, median MCV, tibial MCV, and sural SCV were significantly lower in the mild NPDR group than in the NDR group (*P* = 0.02, *P* = 0.01, and *P* = 0.008; Table 1 and Figures 1(a) and 1(b)).

Multiple logistic regression analysis revealed that sural SCV was an independent factor associated with the presence of mild NPDR (OR: 0.83; 95% CI: 0.71–0.96; *P* = 0.012; Table 2) and that no other clinical findings, including HbA1c or diabetes duration, were associated. Multiple logistic regression analysis also revealed that tibial MCV was an independent factor associated with the presence of mild NPDR (OR: 0.69; 95% CI: 0.51–0.94; *P* = 0.02; Table 3) and that no other clinical findings, including HbA1c or diabetic duration, were associated.

**Figure 2:**
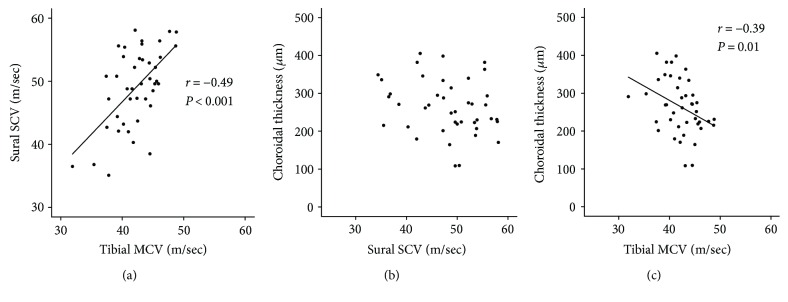
Relationship between nerve conduction velocity and choroidal thickness. (a) Tibial motor conduction velocity (MCV) was positively correlated with sural SCV (*r* = 0.49, *P* < 0.001). (b) There was no relationship between sural sensory conduction velocity (SCV) and choroidal thickness. (c) Tibial MCV was negatively correlated with choroidal thickness (*r* = −0.39, *P* = 0.01).

Tibial MCV was positively correlated with sural SCV (*r* = 0.49, *P* < 0.001, Figure 2(a)). However, there was no such association between sural SCV and CT (Figure 2(b)). By contrast, tibial MCV was negatively correlated with CT (*r* = −0.39, *P* = 0.01, Figure 2(c)).

Finally, multiple regression analysis revealed that age and carotid IMT were independent associated factors with CT (*P* = 0.032 and *P* = 0.009, resp.; Table 4). Multiple regression analysis also revealed that age, tibial MCV, and carotid IMT were independent associated factors with CT (*P* = 0.035, *P* = 0.015, and *P* = 0.008, resp.; Table 5).

## 4. Discussion

We set out to determine associations between ocular and systemic clinical findings, particularly NCV, in type 2 diabetes patients with early DR. We found that sural SCV and tibial MCV were significantly lower in patients with mild NPDR than those with NDR. Furthermore, logistic regression analysis revealed that these types of NCV were independent factors associated with the presence of mild NPDR. In addition, tibial MCV was negatively correlated with CT. Finally, a multiple regression analysis revealed that age, tibial MCV, and carotid IMT were associated with CT.

The association between reduced NCV and the presence of mild NPDR was one of the most interesting findings of the current study. Duration of diabetes and the level of HbA1c have been reported to be the most important risk factors for DR [22, 23]. In the current study, these factors were similar in patients with NDR and mild NPDR, and a multiple regression analysis revealed that while diabetes duration and HbA1c level were not associated with the presence of mild NPDR, peripheral NCV was associated. This finding extends existing knowledge of alterations in peripheral NCV during early diabetes. Clinically, the most frequent and earliest sign of diabetic neuropathy is abnormal plantar sensation, making the association of SCV impairment and DR easy to understand. However, MCV has also been reported to be very useful for the early detection of diabetic polyneuropathy [24, 25]. Previous research has shown that impaired NCV is closely correlated with DR, proteinuria, and the duration of diabetes [10] and that median MCV becomes significantly reduced with the progression of DR [11]. Moreover, reductions in median MCV, as well as SCV, have been reported to be correlated with the progression of DR, from more than 10 years before it first manifests until it reaches the proliferative stage [11]. Unfortunately, we could not confirm previous findings that median SCV was also reduced in NPDR (we did not find a significant difference: *P* = 0.06) [11]. One possible reason for this discrepancy is current limitations in the ability to accurately measure upper limb NCV, which can be affected by factors such as carpal-tunnel syndrome [26]. Furthermore, the nerves in the lower limbs have been reported to be more susceptible than those in the upper limbs to the effects of diabetes, suggesting that longer nerves may be affected more often [27]. Diabetes also generally affects the most distal peripheral nerves earlier. Thus, the lower limbs may be the most appropriate location for accurately measuring impairment of peripheral NCV, including sural SCV and tibial MCV, and we therefore consider that our finding of an association between NCV and DR is reliable.

Another interesting finding in the current study was that tibial MCV was associated with subfoveal CT. Diabetic choroidopathy is thought to originate, like DR, in microangiopathy [28, 29], but the relationship between CT and NCV has not been adequately investigated. Although the significance of changes in CT in eyes with DR still remains the subject of debate, clinical reports have shown that CT increases significantly as the severity of DR progresses through the mild, moderate, and NPDR stages, culminating in PDR [16]. Furthermore, CT decreases after photocoagulation treatment [16]. The subfoveal choroid is also thicker in eyes with DME than without DME, being thickest in eyes with serous retinal detachment-type DME [16]. Other research has shown that patients with early DR, that is, NPDR, had higher CT than healthy controls and that choroidal thickening was more severe in diabetes patients with neuropathy than those without neuropathy [19]. This might be due to the fact that smooth muscle in the choroid is dominated by the autonomic nervous system, which can be impaired in diabetes, that is, in diabetic autonomic neuropathy. Autonomic influences on the vasculature of the eye have been shown to directly control ocular blood flow in the optic nerve, choroid, ciliary body, and iris and to have an indirect influence on retinal blood flow [30]. Contrary to the close relationship between tibial MCV and CT, the current study showed no relationship between sural SCV and CT, a finding that remains hard to explain. This result may have been due to measurement errors in sural SCV. Inadequate control of skin temperature and measurement error of the skin surface distance affect more SCV than MCV [31]. Indeed, the reproducibility of sural SCV is low among several parameters of a nerve conduction study [32]. Thus, the results of previous studies, considered together with our new finding that lower-limb MCV is related to CT, suggest that diabetic autonomic impairment may also be associated with alterations in the choroid, which is dominated by the autonomic nervous system, in eyes with NDR or early DR during the early, impaired glucose tolerance stage of diabetes.

Another novel finding of this study was that carotid IMT was associated with CT in a multiple regression analysis. Ultrasound measurement of IMT reflects the thickness of the tunica intima and tunica media, which are the inner two layers of the artery wall. IMT is clinically used to detect the presence of atherosclerotic disease and to evaluate the progression of atherosclerosis over time [33, 34]. There are numerous clinical reports showing that carotid IMT is associated with type 2 diabetes and that the IMT increases in about one-third of diabetes patients with impaired glucose tolerance [35]. Additionally, clinical reports on the relationship between carotid IMT and DR have shown that carotid IMT is greater in patients with PDR than those with NPDR [36]. Type 2 diabetes-associated carotid plaque burden is also higher in patients with DR than those without DR [37]. Thus, based on current and past results, we speculate that increased carotid IMT, which normally reflects thickening of large vessels such as the carotid artery, is also closely associated with the pathogeneses of both DR and diabetic choroidopathy, diseases associated with alterations in much smaller vessels. These alterations in peripheral microvascular structure are associated with the pathogenesis of diabetes-related complications, even in NDR and early DR.

Our study was somewhat limited by its cross-sectional design, its small sample size, and the lack of healthy control subjects. However, previous data shows that normal NCV is 59.1 m/s for median MCV, 65.2 m/s for median SCV, 47.6 m/s for tibial MCV, and 52.0 m/s for sural SCV [32]. Additionally, although we did not measure macular stratified thickness [13, 14] or use full-field electroretinography in the current study, we consider that reports on the association between findings obtained with these methods and diabetic retinopathy are important [38, 39]. While this study found that there may be a relationship between NCV and CT in eyes with early DR, we cannot draw any more detailed conclusions on the underlying cause of this relationship. Although it is likely that diabetic autonomic impairment may cause increased CT, the mechanism of this effect is likely complicated, and it remains unclear. Nevertheless, we consider that our findings on the relationship of NCV and CT in diabetes patients are reliable, because they are based on carefully selected subjects and clinical findings, including NCV, that were objectively measured. Furthermore, we mainly used lower-limb NCV data, which should have excluded any bias that could have been caused by the measurement of upper-limb NCV. Additionally, since different types of disease have different pathological mechanisms and conditions, we focused only on type 2 diabetes, which has an increasing worldwide prevalence, and used similar methods as in previous objective reports on diabetes.

In conclusion, we found that sural SCV and tibial MCV which were lower in mild NPDR patients than in NDR patients were independent factors associated with the presence of mild NPDR and that tibial MCV was negatively correlated with CT. Moreover, decreased tibial MCV was closely associated with DR and with increased CT in type 2 diabetes patients. Therefore, we believe that NCV is a promising biomarker of DR and may have the potential for use when no professional ophthalmological examination is possible. However, it cannot replace a standard ophthalmological examination. Furthermore, nerve conduction, especially tibial MCV, might be a potential source of novel biomarkers of DR during its early stages. Our findings call for additional investigation to determine whether reduced NCV has a causal relationship with diabetic changes in the structure and function of the eye, including CT.

## Figures and Tables

**Table 1:** Clinical characteristics of diabetes patients without diabetic retinopathy (NDR) and with mild nonproliferative diabetic retinopathy (NPDR).

	All	NDR	Mild NPDR	*P* value
Number of eyes	42	25	17	—
Number of patients	42	25	17	—
Age (years)	54.0 (20.8)	57.0 (16.0)	51.0 (26.0)	0.19
Sex (M : F)	26 : 16	15 : 10	11 : 6	1.00^a^
SBP (mmHg)	125.0 (10.0)	125.0 (10.0)	124.0 (10.0)	0.87
Diabetes duration (years)	5.5 (11.0)	4.0 (9.0)	10.0 (9.0)	0.04
Creatinine (mg/dl)	0.7 (0.3)	0.8 (0.3)	0.7 (0.3)	0.65
Total cholesterol (mg/dl)	185.5 (47.8)	186.0 (50.0)	185.0 (38.0)	0.80
HbA1c (%)	9.2 (1.9)	9.0 (1.8)	10.0 (1.5)	0.19
Carotid IMT	0.8 (0.3)	0.8 (0.2)	0.8 (0.3)	0.89
NCV				
Median SCV (m/sec)	58.9 (8.5)	59.6 (6.0)	56.0 (10.5)	0.06
Median MCV (m/sec)	50.4 (6.0)	53.3 (5.8)	49.2 (2.9)	0.02
Sural SCV (m/sec)	49.6 (8.9)	50.8 (5.1)	47.2 (10.5)	0.008
Tibial MCV (m/sec)	42.5 (4.4)	43.2 (3.7)	41.0 (5.4)	0.01
VA (logMAR)	−0.08 (0.1)	−0.08 (0.08)	−0.08 (0.2)	0.67
SE (diopter)	−1.44 (2.7)	−0.75 (2.9)	−2.00 (3.0)	0.32
IOP (mmHg)	16.0 (4.8)	16.0 (5.0)	17.0 (4.0)	0.30
OCT finding				
CMT (*μ*m)	241.5 (36.5)	234 (26.0)	249 (23.0)	0.01
CT (*μ*m)	265.3 (90.5)	248 (59.5)	291 (111.5)	0.10

NDR = no diabetic retinopathy, NPDR = nonproliferative diabetic retinopathy, SBP = systolic blood pressure, IMT = intima-media thickness, NCV = nerve conduction velocity, SCV = sensory conduction velocity, MCV = motor conduction velocity, VA = visual acuity, SE = spherical equivalent, IOP = intraocular pressure, CMT = central macular thickness, CT = choroidal thickness. Continuous variables are expressed as median (IQR). Unmarked *P* value: Mann-Whitney *U* test; ^a^chi-square test.

**Table 2:** Multiple logistic regression analysis of independent factors associated with mild nonproliferative diabetic retinopathy.

Variable		Adjusted OR (95% CI)	*P* value
Dependent	Independent		
Mild NPDR	Creatinine	2.54 (0.06–109.15)	0.63
	Hemoglobin A1c	0.89 (0.48–1.63)	0.70
	Sural SCV	0.83 (0.71–0.96)	0.012^∗^
	SBP	1.03 (0.99–1.07)	0.16
	Diabetes duration	1.08 (0.95–1.22)	0.24

DR = diabetic retinopathy, SCV = sensory conduction velocity, SBP = systolic blood pressure, NPDR = nonproliferative diabetic retinopathy. Multiple logistic regression analysis; OR: odds ratio; Nagelkerke's *R*-squared = 0.38; ^∗^*P* < 0.05.

**Table 3:** Multiple logistic regression analysis of independent factors associated with mild nonproliferative diabetic retinopathy.

Variable		Adjusted OR (95% CI)	*P* value
Dependent	Independent		
Mild NPDR	Creatinine	1.61 (0.04–67.17)	0.80
	Hemoglobin A1c	0.84 (0.47–1.52)	0.57
	Tibial MCV	0.69 (0.51–0.94)	0.02^∗^
	SBP	1.04 (0.99–1.08)	0.09
	Diabetes duration	1.04 (0.93–1.18)	0.49

DR = diabetic retinopathy, MCV = motor conduction velocity, SBP = systolic blood pressure, NPDR = nonproliferative diabetic retinopathy. Multiple logistic regression analysis; OR: odds ratio; Nagelkerke's *R*-squared = 0.36; ^∗^*P* < 0.05.

**Table 4:** Multiple linear regression analysis of independent factors associated with choroidal thickness.

Variable		*β*	*P* value
Dependent	Independent		
Choroidal thickness	Age	−0.458	0.032^∗^
	Creatinine	−0.149	0.40
	Hemoglobin A1c	−0.176	0.28
	Sural SCV	−0.142	0.41
	Carotid IMT	0.506	0.0086^∗∗^
	Diabetes duration	0.177	0.38

DBP = diastolic blood pressure, SCV = sensory conduction velocity, IMT = intima-media thickness. Multiple linear regression analysis; adjusted *R*-squared = 0.13; *β* = standard partial regression coefficient; ^∗^*P* < 0.05 and ^∗∗^*P* < 0.01.

**Table 5:** Multiple linear regression analysis of independent factors associated with choroidal thickness.

Variable		*β*	*P* value
Dependent	Independent		
Choroidal thickness	Age	−0.396	0.035^∗^
	Creatinine	−0.186	0.26
	Hemoglobin A1c	−0.274	0.078
	Tibial MCV	−0.418	0.015^∗^
	Carotid IMT	0.474	0.0075^∗∗^
	Diabetes duration	0.035	0.86

DBP = diastolic blood pressure, MCV = motor conduction velocity, IMT = intima-media thickness. Multiple linear regression analysis; adjusted *R*-squared = 0.25; *β* = standard partial regression coefficient; ^∗^*P* < 0.05 and ^∗∗^*P* < 0.01.
